# Supplementary material for: Distinct roles for Sir2 and RNAi in centromeric heterochromatin nucleation, spreading and maintenance
Source: EMBO J. 2013 Apr 9;32(9):1250–64. doi: 10.1038/emboj.2013.72 (PMC3642681; doi:10.1038/emboj.2013.72)
Supplement: Supplementary Information [file emboj201372s1.doc]

**Distinct roles for Sir2 and RNAi in centromeric heterochromatin nucleation, spreading & maintenance**

Alessia Buscaino^1^, Erwan Lejeune^1^, Pauline Audergon, Georgina Hamilton, Alison Pidoux and Robin C. Allshire*

**
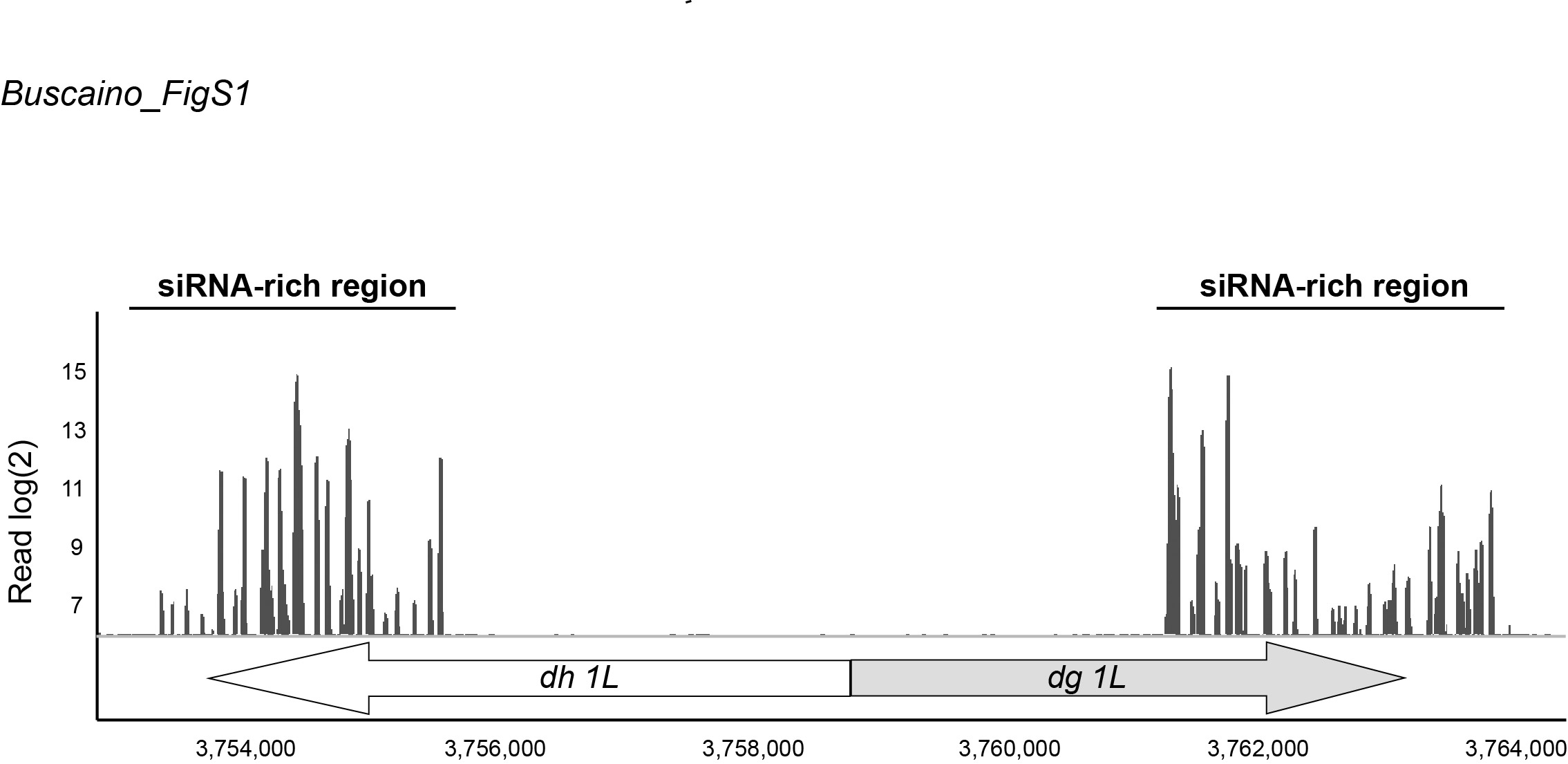
**

**Figure S1**

Sequenced FLAG-Ago1-associated siRNAs from wt cells aligned to the *dh* and *dg* elements of cen1 left. y-axis represent the log2 of the number of sequence reads that align to each nucleotide of the *dh* and *dg* element. x-axis: genome coordinates.


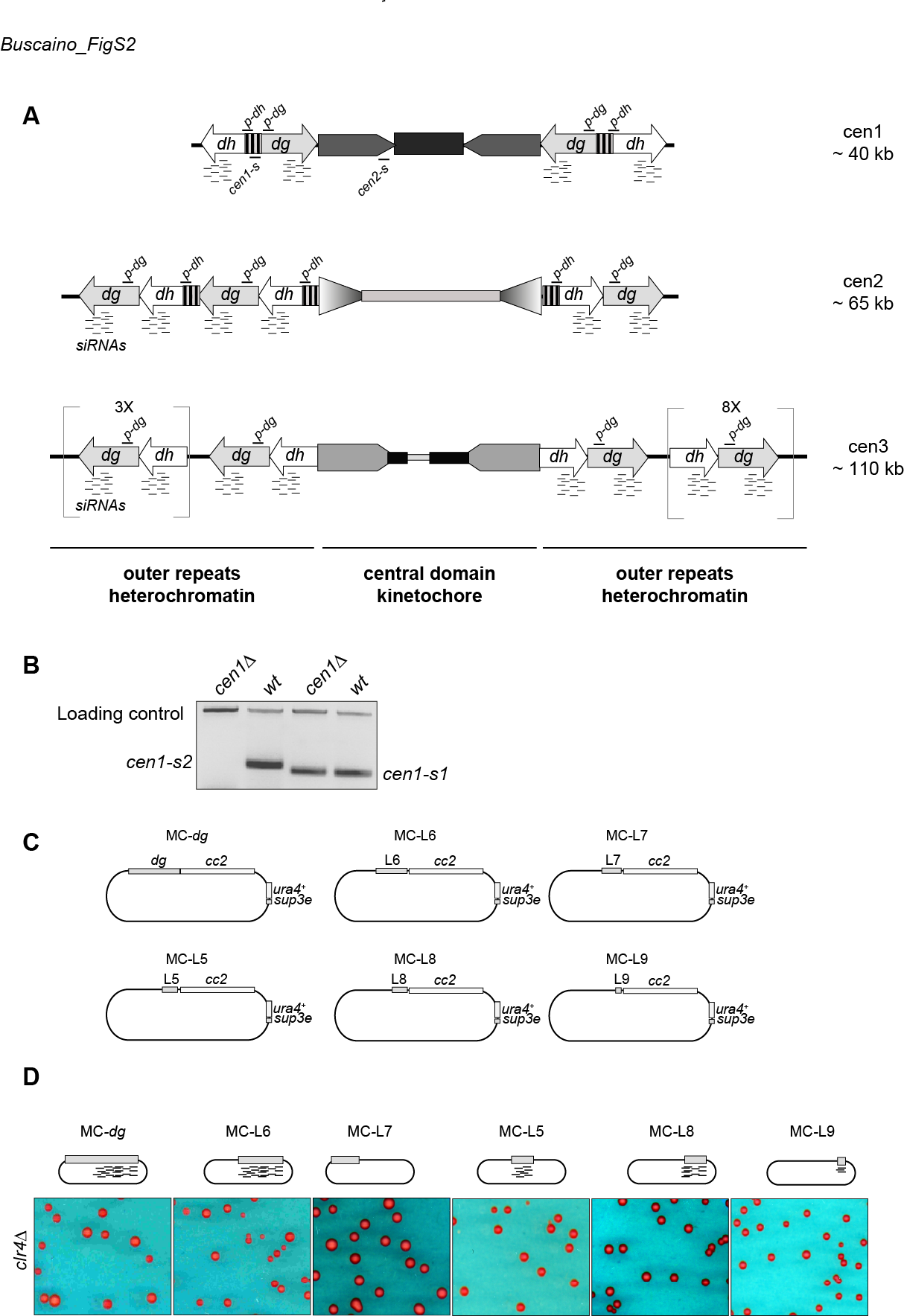


**Figure S2**

1. Diagram of centromere DNA structure. Fission yeast centromeres consist of a central domain where CENP-A^Cnp1^ replaces the histone H3 flanked by outer repeats sequences assembled in heterochromatin. Heterochromatic outer repeats are composed of *dg* (grey arrow) and *dh* (white arrow) elements. Different *dg* and *dh* elements have virtually identical DNA sequences, however their number and organization vary between the three centromeres. A portion of the *dh* element (striped box) is absent at centromere 3. PCR primer pairs (*q-dg* and *q-dh*) hybridize to all three centromeres. Their position relative to the siRNA-rich Nulceation Site is variable and therefore they do not allow discriminating between siRNA-void and siRNA-rich region of *dh* and *dg* elements. PCR Primers cen1-s (heterochromatin-domain) and cen2-s (CENP-A domain), are predicted to allow the amplification of specific products, unique to the *dg*/*dh* arrangement and the central domain at centromere 1 respectively. Adapted from .
2. cen1-s (but not cen2-s) can amplify a PCR product from cells completely lacking cen1 . This suggests that the accepted centromeric repeat sequence and organisation is inaccurate and requires further exploration.
3. Schematic of minichromosomes (MC) used in this study. All minichromosomes used contain, the fission yeast centromeric central domain DNA (cc2) and the *ura4^+^* and *sup3-5* (suppressor of *ade6-704)* selection systems. In addition, MC-dg contains a 5.6kb outer repeat sequence corresponding to the *dg* element (Chromosome 1 coordinates: 3759171-3764746); MC-L6 contains a 3.4kb of *dg* siRNA-rich fragment (Chromosome 1 coordinates: 3761279-3764746); MC-L7 contains a 2.2kb *dg* siRNA-void fragment (Chromosome 1 coordinates: 3759171-3761286); MC-L5 contains 1.6 kb of the *dg* element (Chromosome 1 coordinates: 3761279-3762916); MC-L8 contains 1.6 kb of the *dg* element (Chromosome 1 coordinates : 3763097-3764746); MC-L9 (pLCC9-Fragment J) contains 0.6 kb of the *dg* fragment (Chromosome 1 coordinates: 3762455-3764746).
4. Colony colour assay to assess minichromosome stability in *clr4Δ* cells transformed with minichromosomes bearing *dg*, L6, L7, L5, L8 and L9 fragments were plated on limiting adenine plates to assess minichromosome stability. Red colonies indicate unstable minichromosomes that are lost at mitosis.


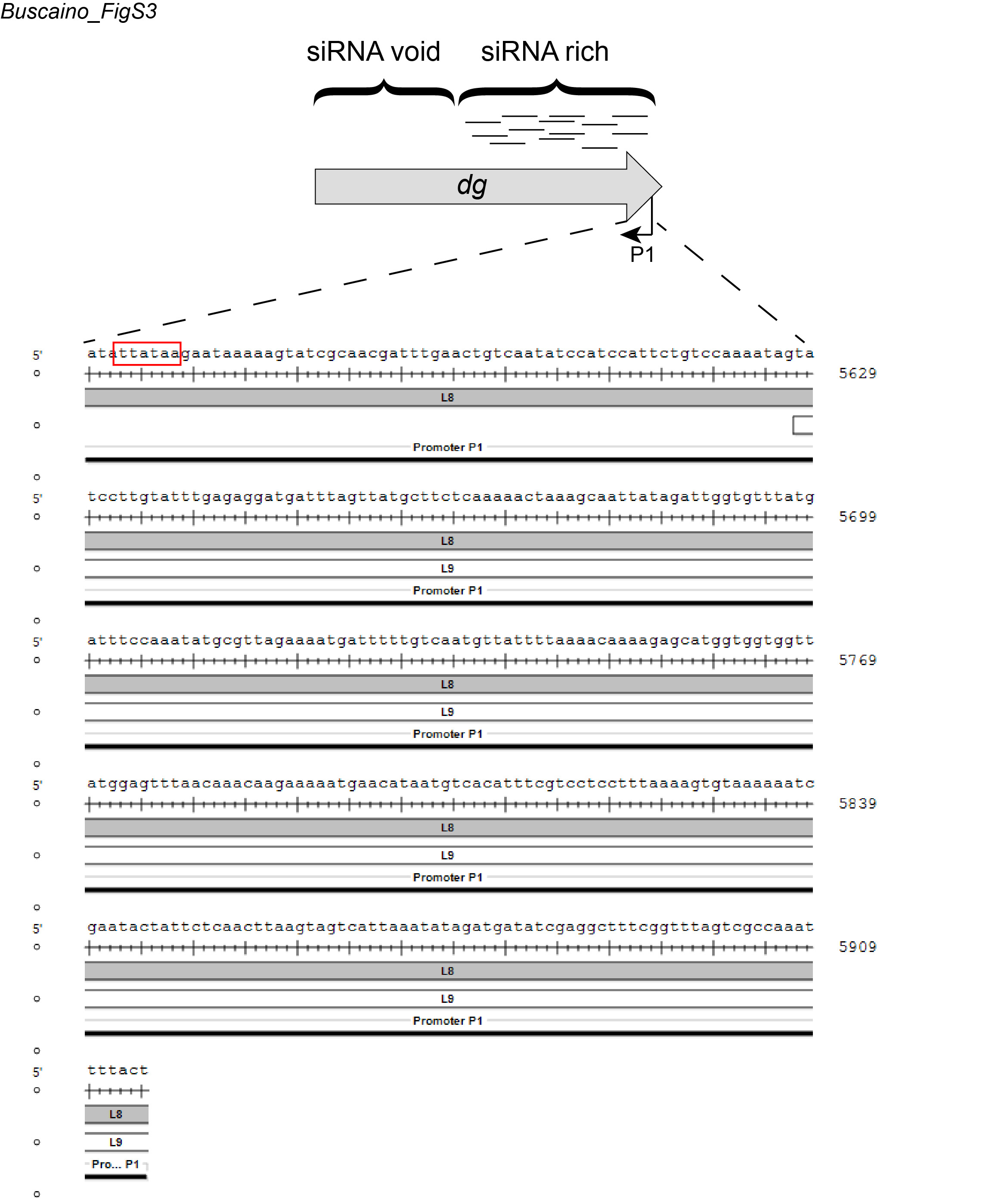


**Figure S3**

Schematic of fission yeast *dg* cen 1 with position of siRNA-rich and void regions. The sequence of the described P1 promoter and the *dg* fragments containing the full promoter (L8) or lacking the TATA box (L9) is shown. TATA box: Red box.

**
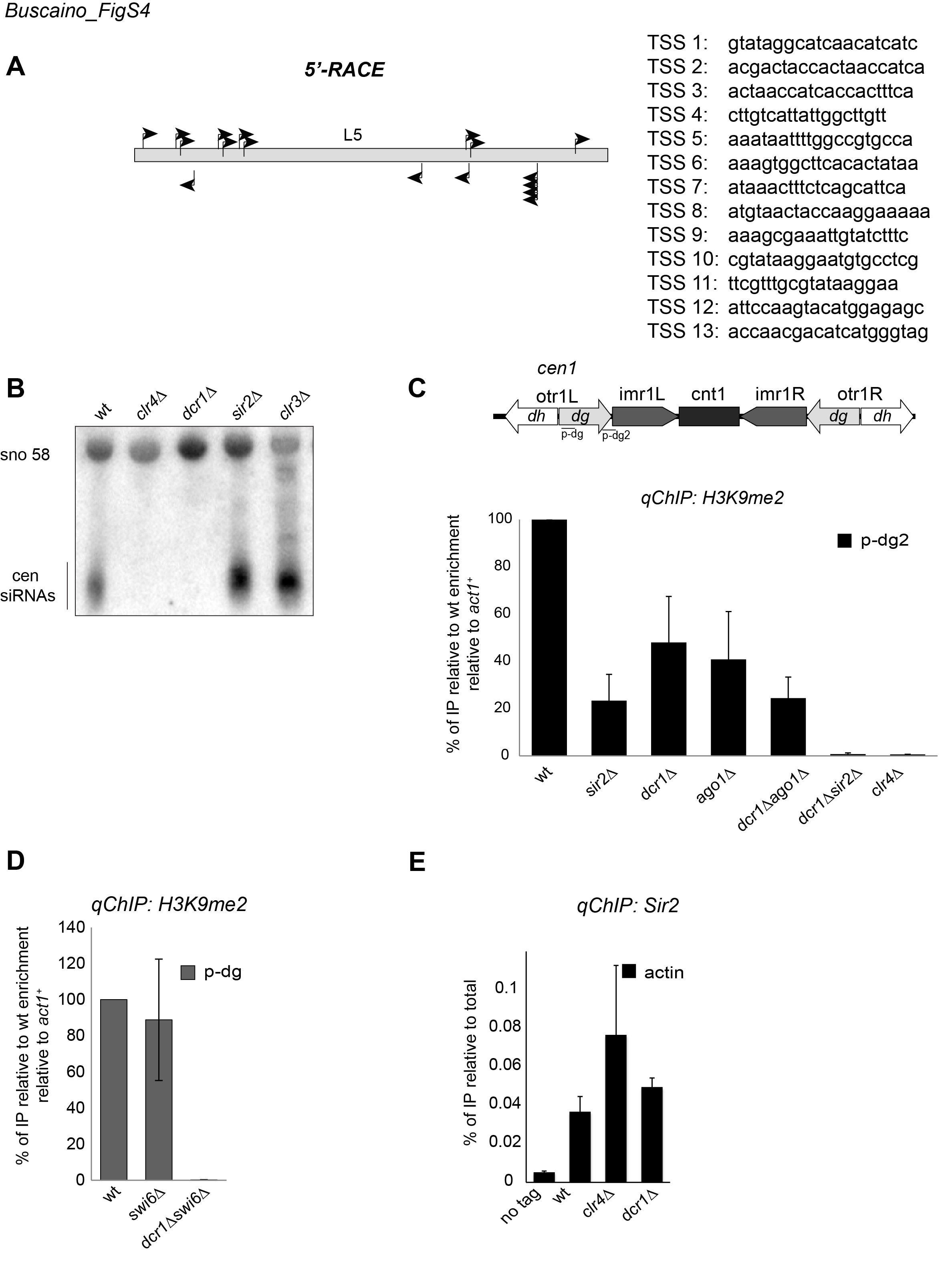
**

**Figure S4**

1. Left: Schematic of L5 transcriptional start sites determined by 5’-RACE-PCR in *clr4Δ* cells Right: 20 bp sequences of each TSS.
2. Northern of centromeric siRNAs in wt, and indicated mutant backgrounds. Loading control: snoRNA58.
3. qChIP analyses of H3K9me2 levels associated with the siRNA-rich region of endogenous centromere *dg* (*p-dg2*) elements. Enrichment is shown relative to total*,* and normalized to wild-type. Error bars: standard deviation of three biological replicates
4. qChIP analyses of H3K9me2 levels associated with endogenous centromere *dg* (*p-dg*) elements in wild-type and indicate mutants. Enrichment is shown relative to total*,* and normalized to wild-type. Error bars: standard deviation of three biological replicates
5. qChIP analyses of FLAG-Sir2 associated with the actin locus in wild-type and indicated mutants background. Error bars: standard deviation of three biological replicates.


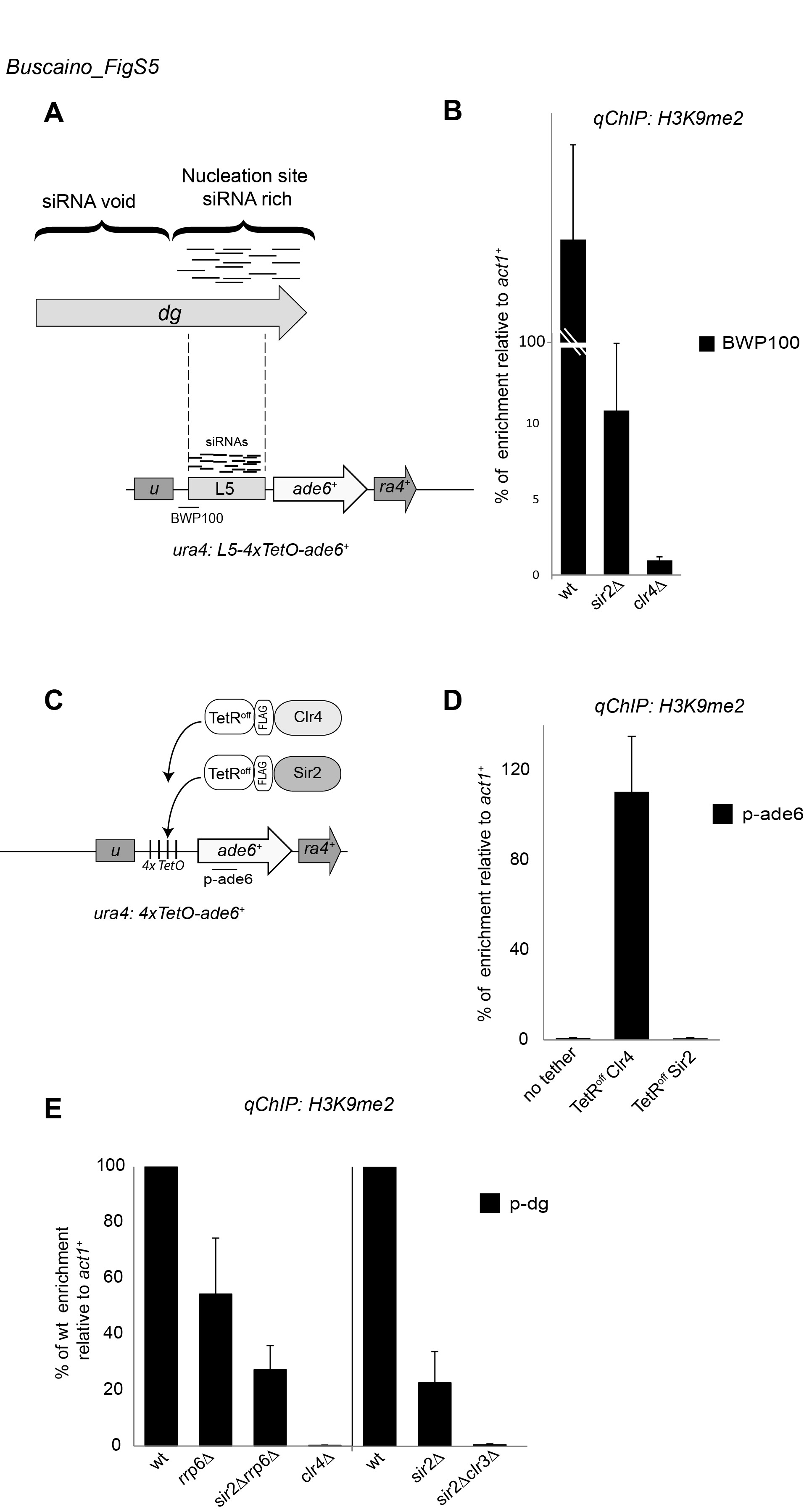


**Figure S5:**

**(A)** Diagram of construct used: The *L5-ade6^+^* reporter is inserted at the *ura4^+^* locus. The position of PCR products (BWP100) on the *L5-ade6^+^* reporter is indicated.

**(B)**  qChIP analyses of H3K9me2 levels associated with *L5-ade6^+^* reporter in wild-type and indicated mutants. Enrichment is shown relative to actin (*act1^+^)*. Error bars: standard deviation of three biological replicates.

**(C)**  Diagram of constructs used: The *L5-4xTetO-ade6^+^* reporter is inserted at the *ura4^+^* locus. TetR^off^-2xFLAG-Sir2 and TetR^off^-2xFLAG-Clr4 are integrated at *leu1^+^* locus. Tethering of TetR^off^-2xFLAG-Clr4 is used as a positive control to monitor heterochromatin establishment at the *L5-4xTetO-ade6^+^* reporter .The position of PCR product (p-ade6^+^) on the *L5-4TetO-ade6^+^* reporter is indicated.

**(D)**  qChIP analyses of H3K9me2 levels associated with *L5-4xTetO-ade6^+^* reporter. Enrichment is shown relative to actin (*act1^+^)*. Error bars: standard deviation of three biological replicates.

**(E)** qChIP analyses of H3K9me2 levels associated with endogenous centromere *dg* (*p-dg*) elements in wild-type and indicated mutants. Enrichment is shown relative to total*,* and normalized to wild-type. Error bars: standard deviation of three biological replicates.

**Table S1. List of strains used in this study**

| **Strain** | **Genotype** | **Figure** |
| --- | --- | --- |
| A2213 | *h^+^ ade6-704:hygMX6 ura4-DSE/D18 leu1-32 his3-D1 cc2:his3^+^ arg3-D4* | Figure 1C, 1D, 1E, 2B, 2C, 4C, S3B, S4B |
| A2207 | *h^90^ clr4Δ::LEU2 ade6-704:natMX6 ura4-DSE/D18 leu1-32 his3-D1 cc2:his3^+^* | Figure 1C, 1D, 1E, 2B, 2C, 4C, S3B, S4B |
| A1508 | *h? sir2Δ::NATMX6 ade6-704 leu1-32 cc2:his3^+^arg3-D4 his3-D1 ura4-D18* | Figure 1E, 2B, 2C, 4C, S3B |
| A7992 | *h? ade6-704:hygMX6 dcr1Δ::KANMX6* | Figure 1E, 2B, 2C, 4C |
| A7857 | *h^90^ ago1Δ::KanMX6 ade6-210 leu1-32 ura4D-18 his3-D1 arg3-D4* | Figure 2B, 2C, 4C |
| A7860 | *h^90^ dcr1Δ::KanMX6 sir2Δ::NatMX6 ade6-210 leu1-32 ura4D-18 his3-D1* | Figure 2B, 2C, 4C |
| 1180 | *h^+^ ade6-210 leu1-32 ura4-D18 otr1R(SphI):ade6^+^* | Figure 3A, 3B, 3C |
| 7411 | *h? cid12-3HA-NatMX6 ade6-210 leu1-32 otr1R(SphI):ade6^+^* | Figure 3A, 3B |
| 8586 | *h? dcr1::NatMX6 otr1(SphI):ade6^+^ ade6-210 ura4-DSE/D18 arg3-D4? his3-D1?* | Figure 3A |
| 8898 | *h^+^ clr4Δ::KanMX6 lys1:NatMX6 otr1R(SphI):ade6^+^ ade6-210 leu1-32 ura4? arg3-D4? his3-D1?* | Figure 3A |
| A7532 | *h^+^ sir2Δ::KanMX6 otr1R(SphI):ade6^+^ ade6-210 arg3-D4? leu1-32? his3-D1? ura4?* | Figure 3A |
| A7533 | *h^-^ cid12-3HA-NatMX6 sir2Δ::KanMX6 otr1R(SphI):ade6^+^ ade6-210 arg3-D4? leu1-32? his3-D1? ura4?* | Figure 3A, 3B |
| A6628 | *h^+^ sir2Δ::KanMX6 ade6-210 leu1-32 ura4-D18 arg3-D4 his3-D1* | Figure 3B |
| 6084 | *h^-^ clr4Δ::LEU2 otr1R(SphI):ura4^+^ ura4-D/SE arg3-D3 leu1-32* | Figure 3B, 3C |
| A7425 | *h^+^ dcr1Δ::KanMX6 otr1R(SphI):ade6^+^ ade6-210 leu1-32 ura4D18* | Figure 3C |
| 8878 | *h^-^ sir2Δ::NatMX6 ade6-210 leu1-32 arg3-D4 his3-D1 ura4-D18* | Figure 3C |
| A7668 | *h^-^ dcr1Δ::KanMX6 sir2Δ::NatMX6 otr1R(SphI):ade6^+^? ade6-210 leu1-32 ura4-D18 arg3-D4? his3-D1?* | Figure 3C |
| 5103 | *h^+^ ars1:(pREP81X)nmt81P_GFP-swi6-LEU2 leu1-32 ura4-D18 ade6-210 his3-D1 arg3-D4* | Figure 4A, 4B |
| 3300 | *h? ars1:(pREP81X)nmt81P_GFP-swi6-LEU2 clr4Δ::ura4 ade6-210 or 216? leu1-32 ura4-DS/E or D18?* | Figure 4A, 4B |
| A7570 | *h? ars1:(pREP81X)nmt81P_GFP-swi6-LEU2 dcr1Δ::KanMX6 ade6-210 arg3-D4? leu1-32 his3-D1? ura4-D18 or DS/E?* | Figure 4A, 4B |
| A7671 | *h? ars1:(pREP81X)nmt81P_GFP-swi6-LEU2 sir2Δ::NatMX6 ade6-210 arg3-D4 leu1-32 his3-D1 ura4-D18* | Figure 4A, 4B |
| A7573 | *h? ars1:(pREP81X)nmt81P_GFP-swi6-LEU2 sir2Δ::ura4 dcr1Δ::KanMX6 otr1R(SphI):ade6 ade6-210 leu1-32 his3-D1 ura4-D18 or DS/E?* | Figure 4A, 4B |
| A8200 | *h^-^ ura4Δ::L5-4xTetO-ade6^+^ leu1^+^:nmt41P_TetOFF-2xFlag-sir2^+^ ade6-DN/N his3-D1* | Figure 5B, 5C, S5 |
| A8277 | *h? ura4Δ::L5-4xTetO-ade6^+^ ade6-DN/N leu1-32 his3-D1? arg3-D4?* | Figure 5B, 5C, S5 |
| A8280 | *h? dcr1Δ::KanMX6 ura4Δ::L5-4xTetO-ade6^+^, ade6-DN/N, leu1-32, his3-D1?, arg3-D4?* | Figure 5B, 5C, S5 |
| A8281 | *h? dcr1Δ::KanMX6 ura4Δ::L5-4xTetO-ade6^+^ leu1^+^:nmt41P_TetOFF-2xFlag-Sir2^+^ ade6-DN/N his3-D1? arg3-D4?* | Figure 5B, 5C, S5 |
| A5498 | *h? clr3Δ:KanMX6 ade6-704:HygMx6 cc2:his3^+^* | Figure S3B |
| A4544 | *h? ade6-704:hygMX6 dcr1Δ::NatMX6 ago1Δ::KanMX6* | Figure S4B |

**Table S2. List of primers used in this study**

| **Primer** | **Sequence** | **Figure** | **Description** |
| --- | --- | --- | --- |
| qACT_FOR | GGTTTCGCTGGAGATGATG | ChIP Figure 1D, 1E, 2C,  4B, 4C, 5C, S3 | Actin control |
| qACT_REV | ATACCACGCTTGCTTTGAG | ChIP Figure 1D, 1E, 2C,  4B, 4C, 5C, S3 | Actin control |
| LS 94 | AATACGACTCACTATAGGGCGAATTG | ChIP Figure 1D | Specific for MC-dg |
| LS 92 | ATCGTCACAGTTTACAAATTCGGT | ChIP Figure 1D | Specific for MC-dg |
|  |  |  |  |
| LS 94 | AATACGACTCACTATAGGGCGAATTG | ChIP Figure 1E, 2C, 3A | Specific for V region |
| LS 92 | ATCGTCACAGTTTACAAATTCGGT | ChIP Figure 1E, 2C, 3A | Specific for V region |
| LS 89 | ATCATTCAGAAAATCACCGGAGCAAT | ChIP Figure 1E, 2C, 3A, 3C, S3 | Specific for R region |
| LS 91 | TCGCCCTAAAAGTAAACGGTAAGC | ChIP Figure 1E, 2C, 3A, 3C, S3 | Specific for R region |
| siRNA left for2 | CAGTCACGACGTTGTAAAAC | ChIP Figure 3A | Specific for A region |
| sirna left rev2 | CTGGTTTGTACTTGCTAAGGG | ChIP Figure 3A | Specific for A region |
| no sirna for2 | CGCTCTAGAACTAGTGGATC | ChIP Figure 3A | Specific for B region |
| no sirna rev2 | CTTGGTGGTAGTTTAATTTGC | ChIP Figure 3A | Specific for B region |
| qPLCC3_rev1 | TGTGGTAGTGGTGCTGTGCTG | ChIP Figure 3A, 3C | Specific for C region |
| qPLCC3_Fw2 | CTCACTATAGGGCGAATTGGA | ChIP Figure 3A, 3C | Specific for C region |
| qPLCC3_fw5 | CTCAATTTTGATCGTATTTCG | ChIP Figure 3A, 3C | Specific for D region |
| qPLCC3_rev4 | GTAAACGGTAAGCACTTTGC | ChIP Figure 3A, 3C | Specific for D region |
| qPLcc7_rev2 | CGGTTCACTTTTCTCTTTC | ChIP Figure 3A, 3C | Specific for E region |
| qPLCC7_Fw3 | CGCTCTAGAACTAGTGGATC | ChIP Figure 3A, 3C | Specific for E region |
| qPLCC9_Rev1 | AACCACCACCATGCTCTTTT | ChIP Figure 3A | Specific for F region |
| qPLCC9_fw2 | CGCGCGTAATACGACTCAC | ChIP Figure 3A | Specific for F region |
| q_dg_FOR | AATTGTGGTGGTGTGGTAATAC | ChIP Figure 4B, 4C, 5C | Specific for all dg fragments in siRNA-void region |
| q_dg_REV | GGGTTCATCGTTTCCATTCAG | ChIP Figure 4B, 4C, 5C | Specific for all dg fragments in siRNA-void region |
| q_dh_FOR | CTACGCTTGATTTGAGGAAGG | ChIP Figure 4C | Specific for all dh fragments in siRNA-void region |
| q_dh_REV | AAAGTATGAGTCGCAGAAGTG | ChIP Figure 4C | Specific for all dh fragments in siRNA-void region |
| AB495 | TAATACGACTCACTATAGGGAGAtcagtactagtgtcagtatag | Northern Figure 5C | Detection of CEN transcripts |
| AB496 | AATTAACCCTCACTAAAGGGAGACCTCTTCGTTTATATCGCTAA | Northern Figure 5C | Detection of CEN transcripts |
| IK8 | ATTCCTTTCTGAACCTCTCTGTTAT | Northern Figure 5E | Detection of CEN siRNAs |
| IK9 | TTTGATGCCCATGTTCATTCCACTTG | Northern Figure 5E | Detection of CEN siRNAs |
| IK10 | GGGAGTACATCATTCCTACTTCGATA | Northern Figure 5E | Detection of CEN siRNAs |
| BWP100F | GGCCTTAGGTAAAAAGCATCG | ChIP Figure 6E | Specific for L5 inserted at ura4 locus |
| BWP100R | TGAGCCCAAGAAGCAATTTT | ChIP Figure 6E | Specific for L5 inserted at ura4 locus |
|  |  |  |  |
| Primer 866 | GAAGAGTTTGAAGACATAGAG | Figure S2 B | cen1-s |
|  |  |  |  |
| Primer 869 | AAGCAATCTAATGAAGGCATG | Figure S2 B | cen1-s |
| DM566 | TTATTGATGGCGAAGCTAGATCCG | Figure S4 C | p-dg2 |
| dg_rev3 | TTTTGCAACCCACATATCGG | Figure S4 C | p-dg2 |
| ade6 for | CATGGAAATTGCAGTGATGG | Figure S5 C, D | p-ade6 |
| ade6 rev | CGAGCAGGGGCATATACTAAA | Figure S5 C, D | p-ade6 |
| WA967 | TACTACTCTAGAGGATCCCATTAGTACCAGTACTAGTG | Cloning pLCC3 |  |
| WA972 | TACTACCTGCAGCCATGGCGATTTTAATATCTGGTGTTG | Cloning pLCC3 |  |
| WA969 | TACTACTCTAGAGGATCCGATAAAGAACTTTTTCCATCCGC | pLCC7 cloning |  |
| WA976 | TACTACCTGCAGCCATGGCCATGGTTTGTTTGTTATATAGAACTAC | pLCC7; pLCC9 cloning |  |
| WA971 | TACTACTCTAGAGGATCCTATCCTTGTATTTGAGAGGATG | pLCC9 cloning |  |

**REFERENCES**

Allshire R. 2003. Centromere and Kinetochore Structure and Function. in *The Molecular Biology of Schizosaccharomyces pombe* (ed. R Egel), pp. 149-169. Spinger

Bayne EH, White SA, Kagansky A, Bijos DA, Sanchez-Pulido L, Hoe KL, Kim DU, Park HO, Ponting CP, Rappsilber J et al. 2010. Stc1: a critical link between RNAi and chromatin modification required for heterochromatin integrity. *Cell* **140**: 666-677.

Djupedal I, Portoso M, Spahr H, Bonilla C, Gustafsson CM, Allshire RC, Ekwall K. 2005. RNA Pol II subunit Rpb7 promotes centromeric transcription and RNAi-directed chromatin silencing. *Genes Dev* **19**: 2301-2306.

Ishii K, Ogiyama Y, Chikashige Y, Soejima S, Masuda F, Kakuma T, Hiraoka Y, Takahashi K. 2008. Heterochromatin integrity affects chromosome reorganization after centromere dysfunction. *Science* **321**: 1088-1091.

Simmer F, Buscaino A, Kos-Braun IC, Kagansky A, Boukaba A, Urano T, Kerr AR, Allshire RC. 2010. Hairpin RNA induces secondary small interfering RNA synthesis and silencing in trans in fission yeast. *EMBO Rep* **11**: 112-118.
